# Denitrifying capability and community dynamics of glycogen accumulating organisms during sludge granulation in an anaerobic-aerobic sequencing batch reactor

**DOI:** 10.1038/srep12904

**Published:** 2015-08-10

**Authors:** Zhang Bin, Xue Bin, Qiu Zhigang, Chen Zhiqiang, Li Junwen, Gong Taishi, Zou Wenci, Wang Jingfeng

**Affiliations:** 1 Institute of Health and Environmental Medicine, Tianjin Key Laboratory of Risk Assessment and Control for Environment and Food Safety, Tianjin 300050, China

## Abstract

Denitrifying capability of glycogen accumulating organisms (GAOs) has received great attention in environmental science and microbial ecology. Combining this ability with granule processes would be an interesting attempt. Here, a laboratory-scale sequencing batch reactor (SBR) was operated to enrich GAOs and enable sludge granulation. The results showed that the GAO granules were cultivated successfully and the granules had denitrifying capability. The batch experiments demonstrated that all NO_3_^−^-N could be removed or reduced, some amount of NO_2_^−^-N were accumulated in the reactor, and N_2_ was the main gaseous product. SEM analysis suggested that the granules were tightly packed with a large amount of tetrad-forming organisms (TFOs); filamentous bacteria served as the supporting structures for the granules. The microbial community structure of GAO granules was differed substantially from the inoculant conventional activated sludge. Most of the bacteria in the seed sludge grouped with members of *Proteobacterium*. FISH analysis confirmed that GAOs were the predominant members in the granules and were distributed evenly throughout the granular space. In contrast, PAOs were severely inhibited. Overall, cultivation of the GAO granules and utilizing their denitrifying capability can provide us with a new approach of nitrogen removal and saving more energy.

The enhanced biological phosphorus removal (EBPR) system is widely accepted as one of the most economical and sustainable processes for removing phosphorus (P) from wastewater^1^,^2^, in which the activated sludge is operated in an anaerobic/aerobi configuration for the enrichment of phosphorusaccumulatingorganisms (PAOs)^3^,^4^. In early studies about EBPR, a group of microorganisms known as glycogen-accumulating organisms (GAOs) were commonly found in reactors with poor EBPR performance^5^. GAOs cannot take up polyphosphate, but they could compete with PAOs for anaerobic volatile fatty acid (VFA) uptake^6^,^7^. Therefore, the enrichment of GAOs is thought to be a major cause of EBPR failure^8^,^9^.

In recent years, granular sludge processes have become an attractive alternative to conventional activated sludge processes for wastewater treatment mainly due to their cell immobilization strategy^10^,^11^,^12^,^13^,^14^,^15^. In addition, the rapid settling velocities of granules have been employed to reduce the size of reactors and could replace large and costly settling tanks^16^,^17^,^18^. In most previous studies, the selection of PAOs and the suppression of GAOs is desired for EBPR^1^,^5^,^7^,^19^,^20^. However, they also need to consume more energy during the aeration phase. Therefore, we proposed to cultivate GAOs granules under limited P conditions to exert their denitrifying function and save more energy. The primary goal of this study was to investigate the denitrifying ability of GAO granules and the ecological characteristics of microbial communities.

In the present study, GAOs were combined with granule processes to cultivate glycogen-accumulating granules (GAGs)^19^. The diverse microorganism populations that coexist in GAGs could uptake organic substances during the anaerobic phase; the most of nutrients were then removed from the bulk liquid before the onset of the aerobic period. Therefore, GAOs can be enriched under these conditions, in favor of other heterotrophic organisms. In the EBPR mode, aeration periods were performed prior to the settling phase and the effluent discharge phase^1^,^6^,^7^, whereas in glycogen-accumulating mode of this study, aeration periods followed the settling and effluent discharge phase. The aeration volume of mixed liquid was decreased to only one-fourth of conventional EBPR system. Therefore, oxygen demand was also greatly reduced. and cultivating GAOs granules could save large amounts of aeration energy. Furthermore, understanding the evolutionary process and distribution characteristics of the microbial community during GAO granulation is important for optimizing of the treatment process. It is also necessary to identify the predominant bacteria in the granules and correlate their population dynamics with process performance.

To address these concerns, the present study was performed to investigate the microbial population dynamics during the development of seeding flocs into granules, as well as the distribution of PAOs and GAOs in different size granules from the sequencing batch reactor (SBR). We demonstrate the evolution and composition of the microbial community from the seeding flocs to granules, and further elucidate the relationship between the distribution of PAOs and GAOs and granule size.

## Results

### SBR performance and granule characteristics

During the start-up period (before day 42), the total organic carbon (TOC) removal efficiency increased gradually (Fig. S1), and was then maintained at >90% after day 47. The biomass demonstrated an enhanced ability for the uptake and degradation of acetate. During reactor operation (~150 days), the total phosphorus content were nearly equivalent in the influent and effluent (Fig. S2), suggesting that the system was incapable of phosphorus removal, and that GAOs were enriched in the reactor.

**Figure 1:**
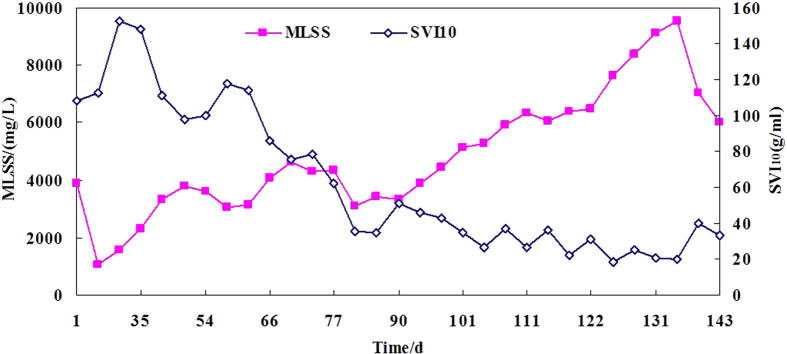
Change in biomass content and SVI_10_ during the whole operation. SVI, sludge volume index; MLSS, mixed liquid suspended solids.

To determine the sludge volume index (SVI) of the granular sludge, a settling time of 10 min was selected rather than 30 min^21^ The SVI_10_ of the inoculating sludge was 108.2 mL g^−1^. The changes in MLSS and SVI_10_ during continuous operation of the SBR are illustrated in Fig. 1. The sludge settleability increased gradually during the set-up and acclimation periods. The SVI_10_ stabilized at 20–40 mL g^−1^, after day 80, which is very low compared with the values measured for conventional activated sludge (100–150 mL g^−1^). The size distribution of the inoculating sludge and granular sludge was measured (Fig. S3), and the results revealed that the mean particle diameter (D[4,3]) of the inoculants was only 54 μm. However, the D[4,3] of the granules that were cultivated in the current study was as high as 340 μm. Moreover, ~60% of the biomass was composed of sludge with particulate matter > 0.2 mm (likely granules).

### Formation and microstructure of granules

**Figure 2:**
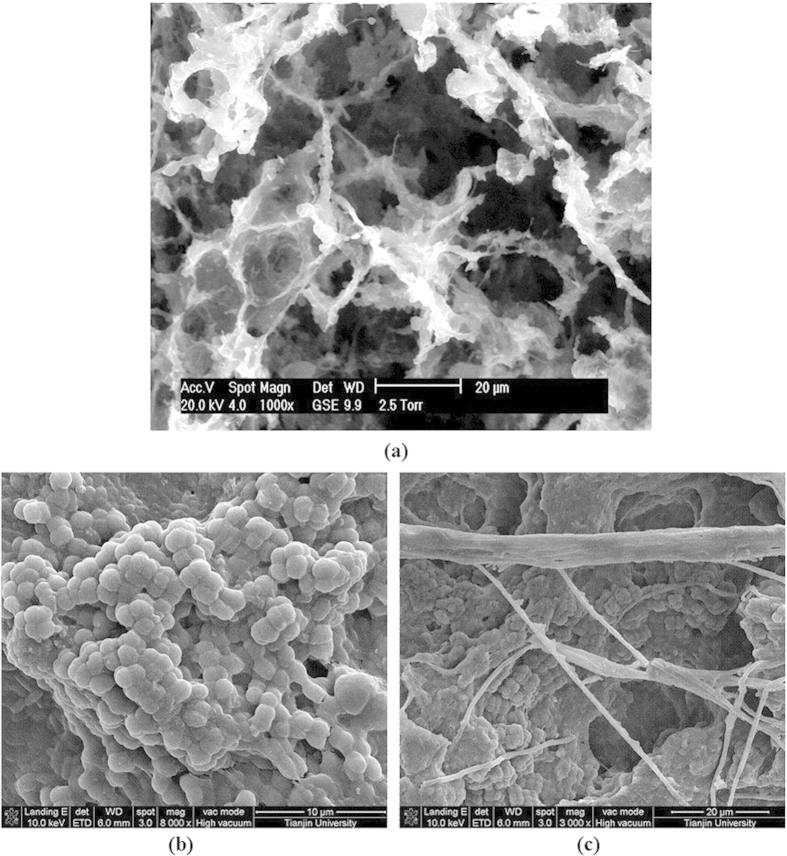
SEM images of the microstructure of seeding sludge (**a**) and granular sludge (**b**,**c**).

To assess the microscale structure of granules, scanning electron microscopy (SEM) was used. As shown in Fig. 2, the structure of inoculants was relatively loose and hollow. However, the granular sludge showed the impacted structure. Images of the granule surface (Fig. 2b) revealed that the surface and core area of the granules were tightly packed with coccoid bacteria, and that a large amount of tetrad-forming organisms (TFOs) were enriched and dominant.

### Microbial population dynamics during GAO granulation

**Figure 3:**
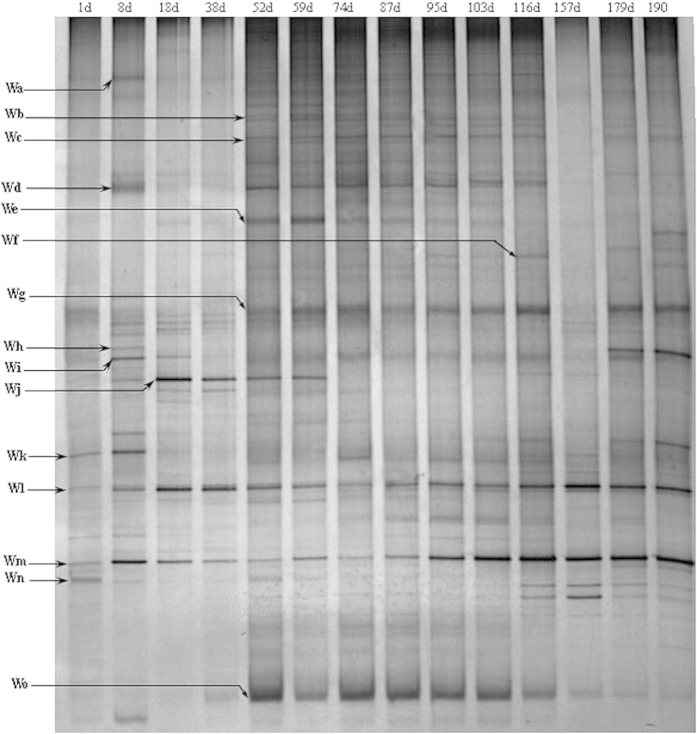
DGGE profile of the bacterial communities in the SBR during the sludge granulation process (labels along the top show the sampling time (days) from bioreactor startup). The major bands are labeled with the numbers (bands Wa to Wo).

**Figure 4:**
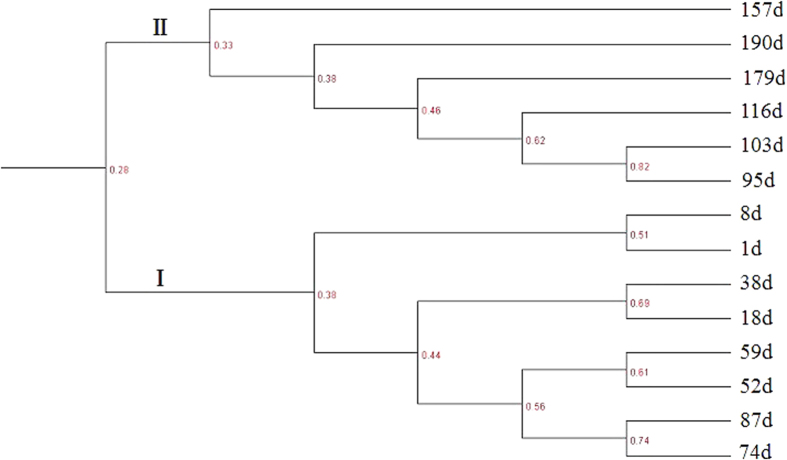
UPGMA analysis of dendrograms of the microbial community DGGE banding patterns showing schematic diagrams of the banding patterns. Roman numerals indicate the major clusters.

Well-resolved denaturing gradient gel electrophoresis (DGGE) bands were obtained at representative points throughout the SBR operation and the patterns revealed the dynamic evolution of the microbial communities during sludge granulation and stabilization (Fig. 3). The community structure at the end of the experiment differed from that of the seed sludge, which suggested that there was a considerable difference in the microbial community structures between the inoculated sludge and enriched GAG sludge. Similarity in the banding pattern was analyzed using UPGMA algorithms (Fig. 4), which revealed two large groups (I and II) with an intragroup similarity of approximately 51-82% and an intergroup similarity of 28%.

The predominant bands shown in Fig. 3 were excised from the gel, and the DNA in the bands was re-amplified, cloned and sequenced. Comparative analysis of the resulting partial 16S rRNA sequences is provided in Table 1. Phylogenetic analysis revealed the phylogenetic affiliation of the 12 sequences retrieved (Fig. S4). Most bacteria in the seed sludge grouped with members of *proteobacterium*.

### Distribution of GAO and PAO in different sized granules

**Figure 5:**
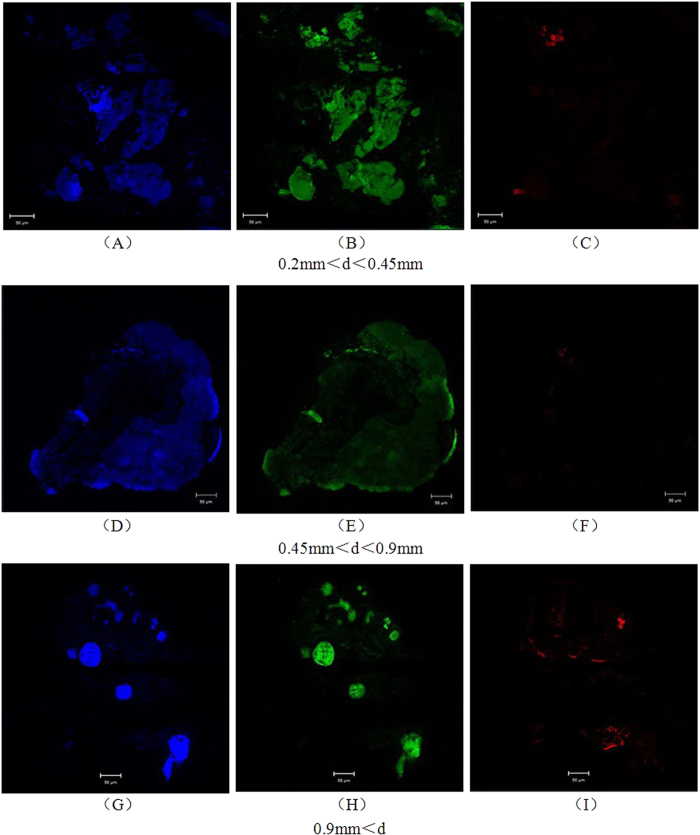
FISH micrographs from experiments performed on three different sized granule sections. DAPI-stained micrographs (**A**,**D**,**G**); GAOs appear as green fluorescence (**B**,**E**,**H**), and PAOs appear as red fluorescence (**C**,**F**,**I**)). Bar = 50μm. d, particle diameter.

FISH was performed on the granule sections to determine the location of GAO and PAO within the different sized granules. As shown in Fig. 5, GAOs were strongly dominant and distributed evenly throughout the granular space, whereas PAOs were inhibited severely and were present only on the edge of granules.

### Denitrifying capability of glycogen-accumulating granules

**Figure 6:**
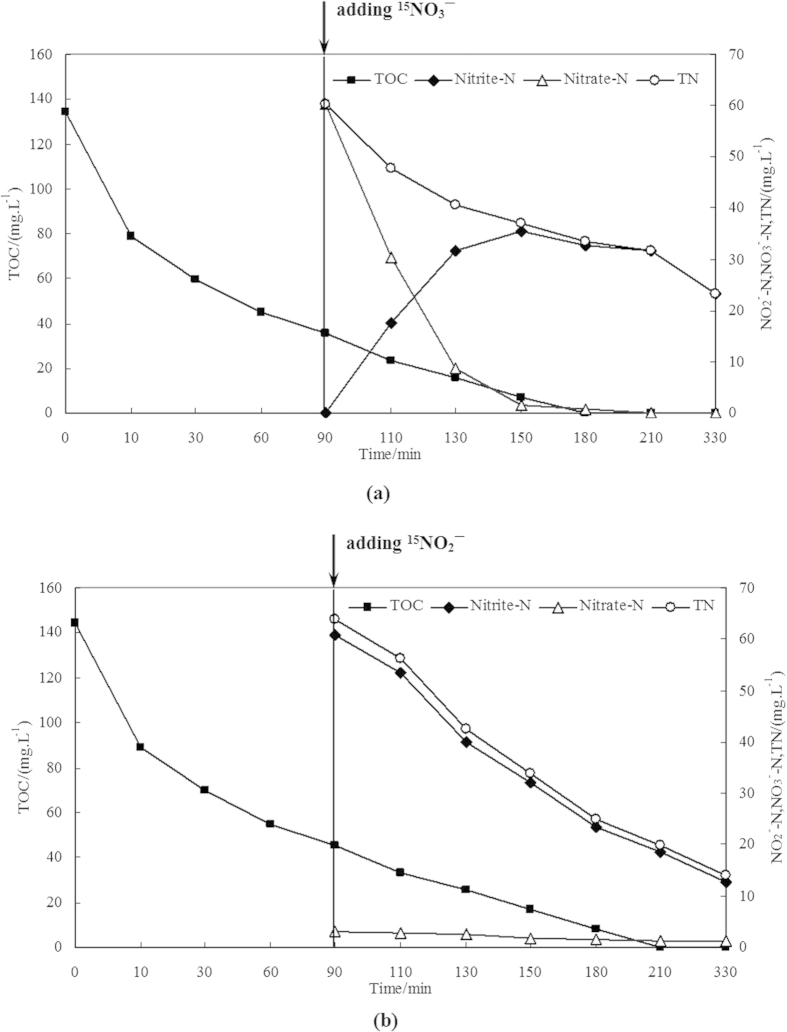
Denitrification by glycogen-accumulating granules with the addition of (**a**) ^15^NO_3_^−^ and (**b**) ^15^NO_2_^−^.

Figure 6a,b shows the results obtained in the batch experiments assessing the denitrifying capability of GAGs for nitrite and nitrate. Most TOCs were absorbed during the anaerobic stirring phase, and nitrate or nitrite reduction occurred immediately after the addition of KNO_3_ or KNO_2_. During the nitrate denitrification testing cycle, the NO_3_^−^-N concentration decreased at a specific reduction rate of 2.5 mg N.(gMLVSS.h)^−1^, wheareas the NO_2_^−^-N concentration increased gradually (Fig. 6a) during system operation. At the end of the batch experiment, all KNO_3_ was removed or reduced, and NO_2_^−^-N had accumulated in the reactor, the removal rate of total nitrogen reached 61%. For nitrite denitrification, the specific reduction rate was ~2.0 mg N.(gMLVSS.h)^−1^, and the removal efficiency of total nitrogen was ~78%. Mass spectroscopy analysis of the gaseous products present during in the denitrification process is shown in Fig. S5. The value of δ^15^N was 1155% in Fig. S5(a) and 1533% in Fig. S5(b), and no other gas peaks were identified, suggesting that N_2_ was the main gaseous product during denitrification.

## Discussion

### GAG cultivation and denitrification

A short settling time was used for granule formation (so-called selecting pressure), and only particles with a settling velocity >4.5 m h^−1^ were retained in the reactor. Therefore, a mass of inoculants were washed out in the start-up phase, and the MLSS content decreased remarkably (from 3876 mg.L^−1^ to 866 mg.L^−1^). Subsequently, biomass with excellent settling ability proliferated, and the MLSS content increased gradually during SBR operation. These finding suggest that the granular sludge was formed according to a microbial self-aggregating process.

Because almost all of the acetate was consumed in the anaerobic phase (feast phase) by GAOs, no external carbon source was available during the aeration period for heterotrophic denitrifiers. Therefore, the presence of ordinary denitrifying heterotrophs was minimized and denitrification was accomplished by denitrifying GAOs (DGAOs) inside the granules, which used intracellular PHB as an electron donor during the starvation period (famine phase)^22^. In the PAOs granule systems, aeration was necessary for P-removal. Therefore, the total nitrogen removal efficiency was lower than in the GAO granule system.

Granules form a range of sizes and structures as a result of variable operational conditions and influences^13^,^15^. In recent studies, some TFOs with a GAO phenotype were reported in laboratory-scale reactors^23^. As shown in the cross section of the granules (Fig. 2c), rod-like and filamentous bacteria were tightly linked together in the inner core of the particle and that coccoid bacteria entered the granules to grow. These finding suggest that rod-like and filamentous bacteria form the supporting structures for granule growth during GSBR processes.

### Relationship between microbial communities dynamic and sludge granulation

Different molecular techniques have been used to elucidate key metabolic aspects of pollutant degradation-related microorganisms^13^,^24^. Many of these are not quantitative, but can help to clarify specific metabolic capacities. Most of the commonly used methods to date have revealed the phylogeny of the populations present in a culture based on the 16S rDNA or rRNA (e.g. FISH or DGGE) or on a functional gene (e.g., qPCR). This information can then be used to link the characteristics of microbes to an observed performance,^17^,^25^. As shown in Fig. 3, a noticeable shift in the microbial population occurred during the start-up phase (before day 52). This was similar to what we reported previously during PAO granulation. The current study used a short settling time for granule formation, and only particles with a settling velocity >4.5 m h^−1^ were retained in the reactor. During this phase, large amounts of biomass could not survive in the reactor and a clear shift in the populations was evident. After day 59, the structure of the microbial community became stable, and more bands were present in the profile due to the improved sludge settling ability and retention of more biomass. Furthermore, mild changes in the intensities of the bands indicated that the microbial communities changed slowly throughout the granulation process. These results suggest that the short settling time (selection pressure) stressed the biomass, leading to a remarkable change in the microbial communities. The UPGMA clustering generally followed the time course of sludge granulation. Group I was associated with start-up phase, washout and decreasing SVI_10_, whereas group II was associated with sludge granulation and excellent biomass settling. Therefore, under the SBR operational conditions, granulation likely leads to the enrichment of microorganisms that contribute to the accumulation of glycogen and granule formation.

The microbial communities could be divided into two main groups according to the origin of highly homologous bacteria. Some were laboratory-scale reactor or large-scale processes with enhanced phosphate removal efficiency, such as bands W_a_, W_d_, W_f_, W_g_ and W_o_. In contrast, others were anaerobic digesters of sludge, such as bands W_e_, W_k_, W_m_ and W_n_. The sequencing results revealed that the operational mode used in this study could provide a more suitable environment for the growth of organisms that could efficiently assimilate substances under anaerobic conditions. Furthermore, some species from the phosphorus removal bioreactor were identified, which suggests that some genera have two different metabolic mechanisms that enable growth in different environment. Most of the bacteria grouped with members of *proteobacterium*. Some species were dominant in the seed sludge and granules, such as *Rhodocyclaceae*, which anaerobically synthesize PHA and aerobically accumulate polyphosphate in EBPR systems^17^,^26^,^27^. *Rhodocyclaceae* are also known for having the ability to produce extracellular polymers (EPS) and serve as the functional strains for aerobic sludge granulation^28^. The dominant species identified in the current study might be useful for inoculating cultures in future studies to jump-start or improve bioreactor performance. Some species that were dominant in the inoculating phase such as *Thauera* sp. disappeared or weakened gradually. *Thauera* are heterotrophic denitrificans found in nitrifying-denitrifying activated sludge and other wastewater treatment systems. In the current study, almost all acetate was adsorbed by GAOs in the anaerobic phase, and no excess carbon source could be provided to the heterotrophic denitrifiers for traditional denitrification after nitrate or nitrite was added.

### Effect of varying in particle size on the distribution of PAOs and GAOs in the granules

Previous investigations of the distribution of PAO and GAO bacterial communities have focused on flocculent activated sludge^24^,^29^. In the present study, different sized granules were sieved, and the effects of granule size on the distribution of biomass species were assessed using visible experimental data, particularly in the single granular sludge reactors. PAOs were scarce primarily because the accumulation of intracellular phosphorus was inhibited in the anaerobic-discharge- aerobic operational mode used in this study; the proportion of PAOs increased only slightly in larger granules (d > 0.9 mm). The larger granules could provide increased space for microbial multiplication, whereas the outer spaces were relatively looser. Accordingly, residual phosphorus in the mixed liquid after the discharge phase could penetrate preferentially into the granules with less restriction, which would favor the proliferation and reproduction of PAOs.

### Engineering application prospect of PAO granules

In this study, the effective aeration volume was only 1 L at aeration phase, whereas in conventional polyphosphate-accumulating mode, the effective aeration volume was about 4L. Obviously, comparing with cultivating PAO in EBPR mode, cultivating GAO granules could save more aeration energy while maintaining the same concentration of dissolved oxygen during aeration periods. The results of the research will provide new ideas and theoretical support to improve existing conventional biological wastewater treatment process. Especially, for SBR process, cultivating GAO granules only need to change the operating sequence of original reactor, without the need for new treatment plants. Subsequently, the original aerobic phase effluent could be mixed with PAO granules, and the denitrifying would be achieved.

In conclusion, the GAO granules were cultivated successfully to have some denitrifying capability, resulting in the production of N_2_ as the main gaseous product. In this labscale experiment, camparing with polyphosphate-accumulating mode, cultivating GAO granules could save aeration energy. GAOs were the dominant species and were distributed evenly throughout the granular space, whereas the growth of PAOs was inhibited severely. Future work can apply these methods to pilot-scale experiment to explore the feasibility of improving conventional wastewater treatment systems.

## Methods

### Reactor set-up and operation

Granules were cultivated in a lab-scale SBR with an effective volume of 4 L. The effective diameter and height of the reactor were 10cm and 50cm, respectively. The hydraulic retention time was set at 8 h. Activated sludge from a full-scale sewage treatment plant (Jizhuangzi Sewage Treatment Works, Tianjin, China) was used as the seed sludge for the reactor at an initial sludge concentration of 3876 mg L^−1^ in the MLSS. In the laboratory , the reactor was operated on 6-h cycles consisting of a 2-min influent feeding phase, a 90-min anaerobic phase (mixing), a (5 ~ 10)-min settling phase, a 5-min effluent discharge phase and 240-min aeration periods. Under these conditions, GAOs could be enriched over other heterotrophic organisms because of their ability to uptake acetate under anaerobic conditions^30^. In the polyphosphate-accumulating mode^31^, aeration periods were performed prior to the settling phase and the effluent discharge phase, whereas aeration periods followed the effluent discharge phase in glycogen-accumulating mode. The volume of mixed liquid was decreased to only one-fourth of its original level after effluent discharge phase. The airflow rate was set to 0.5-0.8 L.min^−1^. The sludge settling time was gradually reduced from 10 to 5 min after 80 SBR cycles in 20 days, and only particles with a settling velocity higher than 4.5 m h^−1^ were retained in the reactor. The compositions of the influent media were NaAc (450 mg L^−1^), NH_4_Cl (100 mg L^−1^), (NH_4_)_2_SO_4_ (10 mg L^−1^), KH_2_PO_4_ (20 mg L^−1^), MgSO_4_.7H_2_O (50 mg L^−1^), KCl (20 mg L^−1^), CaCl_2_ (20 mg L^−1^), FeSO_4_.7H_2_O (1 mg L^−1^), pH 7.0-7.5, and 0.1 mL L^−1^ trace element solution.

### Analytical methods

The levels of total organic carbon (TOC), NH_4_^+^-N, NO_2_^−^-N, NO_3_^−^-N, total phosphate (TP), mixed liquid suspended solids (MLSS), and the sludge volume index at 10 min (SVI_10_) were measured regularly according to standard methods^32^ The particle size distribution of the seeding sludge and granules was determined using a laser particle size analyzer (Malvern Mastersizer, Malvern Instruments Ltd, UK).

The microstructure of the inoculants and granules in the reactor was observed using scanning electron microscopy (Nanosem 430, FEI Inc., USA). Samples for SEM were fixed in 2.5% glutaraldehyde in a 25 mM cacodylate buffer at pH 7.0. Further sample preparations and microscopic analyses were conducted as described previously^31^.

Denitrification batch experiments and the detection of gaseous products were performed at the end of the anaerobic period. A stable isotope ^15^N-labeled KNO_3_ or KNO_2_ was added to obtain an initial nitrogen concentration of 60 mgN L^−1^. Subsequently, the anoxic period was maintained and stirring continued. Sampling was performed at different time points, and the TOC and NO_x_-N were examined. Gaseous products were collected and analyzed using isotopic mass spectrometry. Each batch test was repeated three times.

### DNA extraction and polymerase chain reaction-denaturing gradient gel electrophoresis (PCR-DGGE)

The sludge from approximately 8 mg of MLSS was transferred into a 1.5-ml Eppendorf tube and then centrifuged at 14,000 g for 10 min. The supernatant was then removed, after which the pellet was added to 1 ml of sodium phosphate buffer solution and aseptically mixed with a sterilized pestle to detach granules. Genomic DNA was extracted from the pellets using an E.Z.N.A.^TM^ Soil DNA kit (D5625-01, Omega Bio-Tek Inc., USA).

Subsequently, the variable V3 region of the bacterial 16S rRNA gene sequence (corresponding to positions 341–534 of the Escherichia coli sequence) was then PCR-amplified using a PCR Authorized Thermocycler (Eppendorf, Hamburg, Germany) according to a method established previously^33^.

The PCR amplicons were separated by DGGE on polyacrylamide gels (8%, 37.5:1 acrylamide/bisacrylamide) with a linear gradient of 35- 60% denaturant (100% denaturant = 7 M urea plus 40% formamide). The gel was run for 7 h at 130 V in 1 × TAE buffer (40 mM Tris-acetate, 20 mM sodium acetate, and 1 mM Na_2_EDTA, pH 7.4) and maintained at 60 °C (DCode^TM^ Universal Mutation Detection System, Bio-Rad, CA, USA). Following electrophoresis, the gels were silver-stained and developed^34^, and were then scanned using a gel imaging analysis system (Image Quant350, GE Inc., USA). Dendrograms that assessed the similarities of band patterns were calculated automatically without band weighting (consideration of band density) using the unweighted pair group method with arithmetic mean (UPGMA) algorithms available in the Quantity One Software, version 4.31 (Bio-Rad).

Prominent DGGE bands were excised and dissolved in 30 μL Milli-Q water overnight at 4 °C. The target DNA fragments were then cloned and sequenced using an established method^31^.

### Distribution of PAOs and GAOs

Three size classes of granules (0.2–0.45, 0.45–0.9, and >0.9 mm) were selected on day 180 for fluorescence *in situ* hybridization (FISH) analysis to assess the spatial distribution characteristics of PAOs and GAOs in granules. FISH was then performed as described previously^35^. The 16S rRNA-targeted oligonucleotide probes used for the *in situ* detection of PAOs and GAOs were Cy5-labeled PAOMIX (PAO462, PAO651 and PAO846) specific for ‘*Candidatus* Accumulibacter phosphatis’^36^, FITC-labeled GAOQMIX (GAOQ431 and GAOQ989) specific for ‘*Candidatus* Competibacter phosphatis’^36^, FITC-labeled DF1MIX (TFO_DF218, TFO_DF618 and TFO_DF862) for Cluster 1 of *D.* vanus-related *Alphaproteobacteria*^29^, and FITC-labeled DF2MIX (DF988 and DF1020) with helper probes (H966 and H1038) for Cluster 2 of DvGAOs^24^. All probes were used with a formamide concentration of 35%. Finally, 1 ng.μL^−1^ 4,6-diamidino-2-phenylindole (DAPI) was used to detect all DNA. The hybridized images were then captured using a confocal laser scanning microscope (CLSM, Zeiss 710).

## Additional Information

**How to cite this article**: Bin, Z. *et al.* Denitrifying capability and community dynamics of glycogen accumulating organisms during sludge granulation in an anaerobic-aerobic sequencing batch reactor. *Sci. Rep.* **5**, 12904; doi: 10.1038/srep12904 (2015).

## Supplementary Material

Supplementary Information

## Figures and Tables

**Table 1:** Species identification in the selected DGGE bands (the bands are labeled in Fig. 5)^a^.

Bands	Closest relatives (Accession no.)	Identity(%)	Origin
Wa	Uncultured *gamma proteobacterium* (HQ341388)	97	activated sludge from a phosphorus removal bioreactor
Wd	Uncultured *bacterium* (FJ356055)	96	anaerobic:aerobic lab-scale EBPR activated sludge system
We	Uncultured *bacterium* (FJ534989)	100	anaerobic fermentation reactor with the mixture of waste activated sludge and carbohydrate
Wf	Uncultured *bacterium* (HQ010712)	98	anaerobic/aerobic/anoxic multistage sequencing batch reactor with the carbon source of acetic acid for nitrogen and phosphorus removal
Wg	Uncultured Rhodocyclaceae bacterium (GU123151)	99	activated sludge from a wastewater treatment plant which was operated with an anaerobic (1h)-aerobic (4h) process
Wh	Uncultured *bacterium* (HM596318)	95	activated sludge cultivated to produce PHAs
Wj	Uncultured Thauera sp. (GU257672)	98	activated sludge in a membrane bioreactor
Wk	Thiothrix sp. (JF824663)	100	bacterial mat
Wl	Uncultured *bacterium* (FJ623321)	98	aerobic sequencing batch reactor under aerobic feeding and discharge conditions with the carbon source of waste activated sludge alkaline fermentation liquid
Wm	Uncultured Rhodocyclaceae bacterium (HQ003474)	97	Carrizo shallow lake
Wn	Uncultured *beta proteobacterium* (CU918807)	96	mesophilic anaerobic digester which treats municipal wastewater sludge
Wo	Uncultured *gamma proteobacterium* (GU066593)	100	polyphosphate-accumulating granular sludge

^a^The partial sequences of the 16S rRNA genes obtained in this study were submitted to the GenBank database under accession numbers JQ280451-JQ280451.
