# Supplementary material for: Denitrifying capability and community dynamics of glycogen accumulating organisms during sludge granulation in an anaerobic-aerobic sequencing batch reactor
Source: Sci Rep. 2015 Aug 10;5:12904. doi: 10.1038/srep12904 (PMC4530441; doi:10.1038/srep12904)
Supplement: Supplementary Information [file srep12904-s1.doc]

Denitrifying capability and community dynamics of glycogen accumulating organisms during sludge granulation in an anaerobic-aerobic sequencing batch reactor[[1]](#footnote-2)

Zhang Bin , Xue Bin, Qiu Zhigang, Chen Zhiqiang, Li Junwen, Gong Taishi, Zou Wenci & Wang Jingfeng*

Institute of Health and Environmental Medicine, Tianjin Key Laboratory of Risk Assessment and Control for Environment and Food Safety, Tianjin 300050, China.

**Supporting Information**

Figure S1︱Long-term influent and effluent TOC concentrations showing the acetate removal performance of the SBR. TOC, total organic carbon.

Figure S2︱Long-term influent and effluent TP concentration during the SBR operation. TP, total phosphate.

Figure S3︱Particle size distribution in the seeding sludge (A) and GAO granular sludge (B)

Figure S4︱Phylogenetic tree based on a comparison of 16S rDNA sequences from predominant bacteria in the biomass. The scale bar represents 5% estimated sequence divergence.

Figure S5︱Mass spectroscopy detection result of denitrifying gaseous product (a) with ^15^NO_3_**^—^** addition and (b) with ^15^NO_2_**^—^** addition.

Figure S1︱Long-term influent and effluent TOC concentrations showing the acetate removal performance of the SBR. TOC, total organic carbon.

Figure S2︱Long-term influent and effluent TP concentration during the SBR operation. TP, total phosphate.


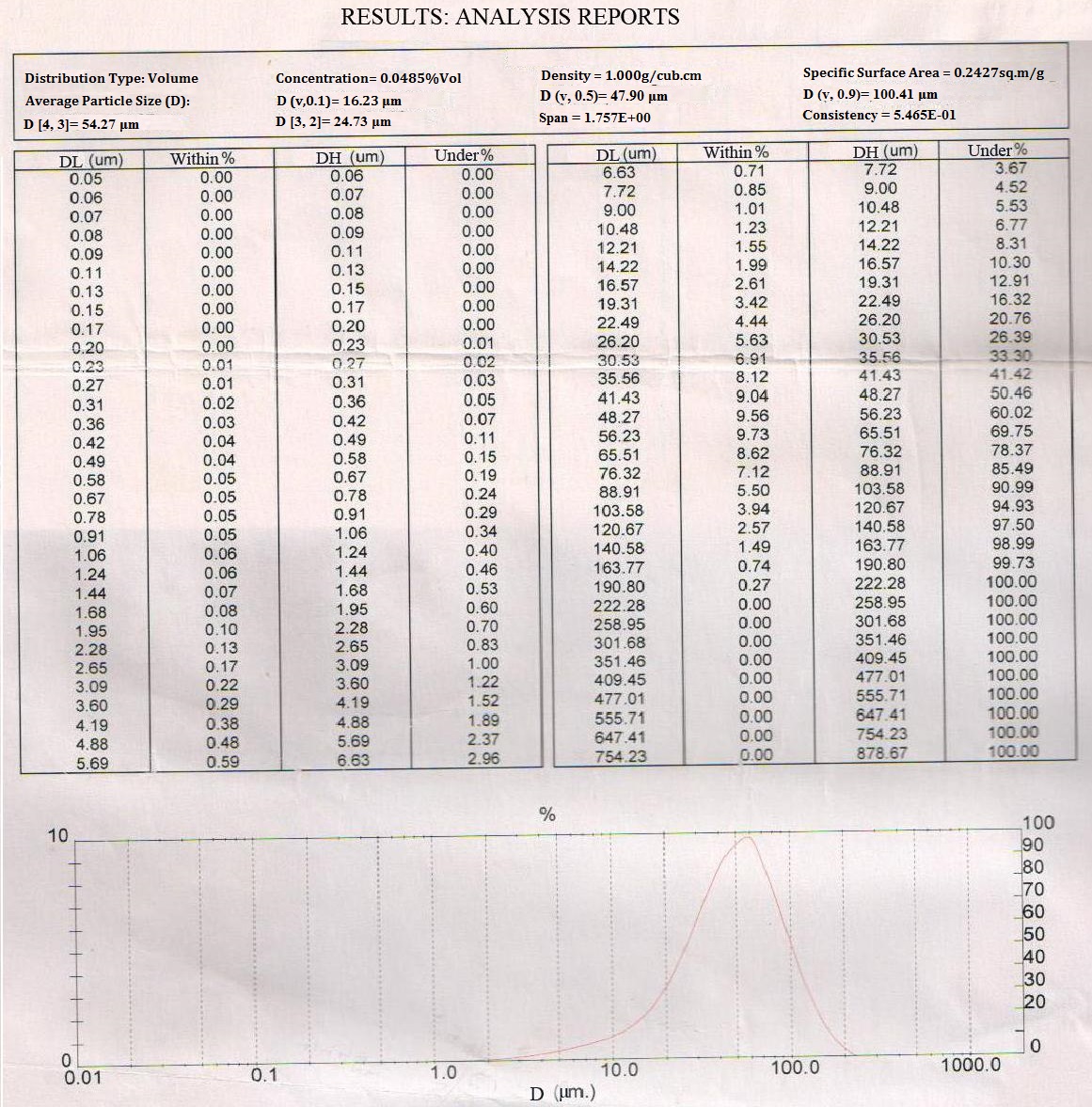


**(A)**


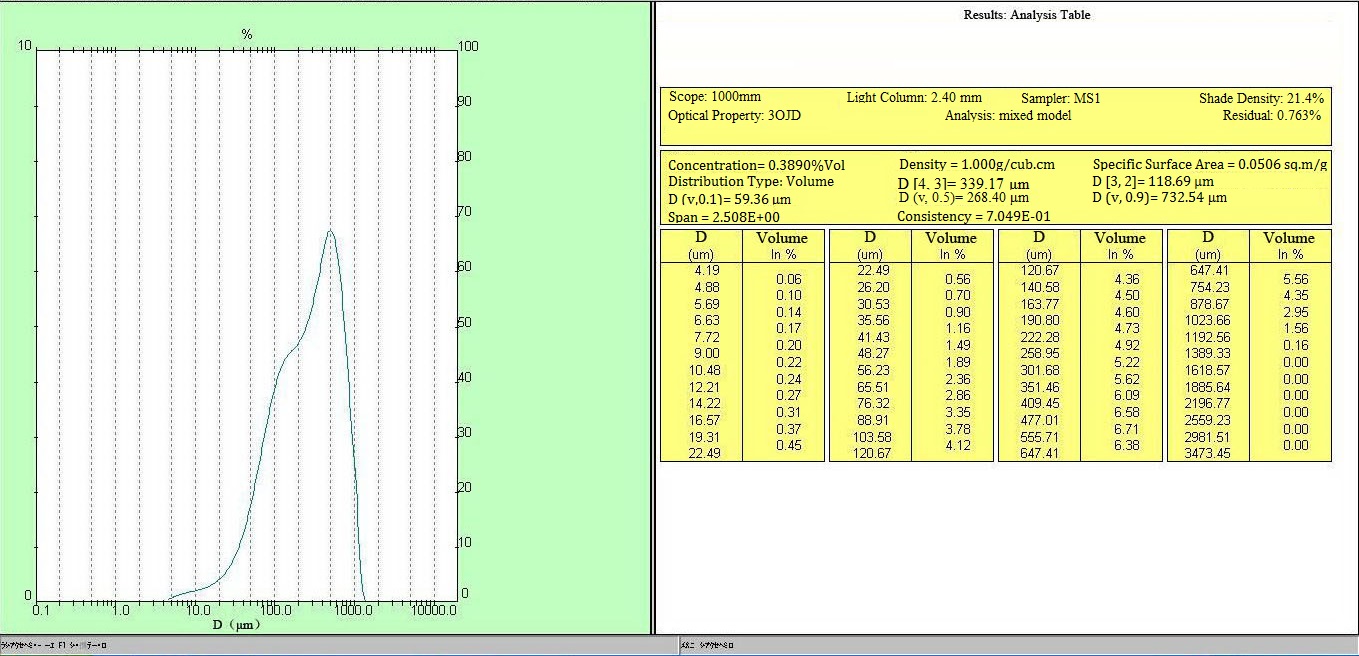


**(B)**

Figure S3︱Particle size distribution in the seeding sludge (A) and GAO granular sludge (B)


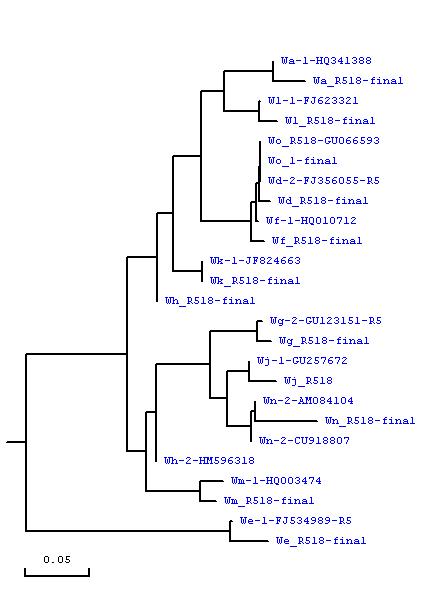


Figure S4︱Phylogenetic tree based on a comparison of 16S rDNA sequences from predominant bacteria in the biomass. The scale bar represents 5% estimated sequence divergence.

Uncultured *gamma proteobacterium*(HQ341388)

Band_Wa

Uncultured *bacterium* (FJ623321)

Band_Wl

Uncultured *gamma proteobacterium* (GU066593)

Band_Wo

Uncultured *bacterium*(FJ356055)

Band_Wd

Uncultured *bacterium*(HQ010712)

Band_Wf

*Thiothrix sp.* (JF824663)

Band_Wk

Band_Wh

Uncultured *Rhodocyclaceae bacterium* (GU123151)

Band_Wg

Uncultured *Thauera* sp. (GU257672)

Band_Wj

*Thauera* sp. (AM084104)

Band_Wn

Uncultured *beta proteobacterium*(CU918807)

Uncultured *bacterium*(HM596318)

Uncultured *Rhodocyclaceae bacterium* (HQ003474)

Band_Wm

Uncultured *bacterium* (FJ534989)

Band_We


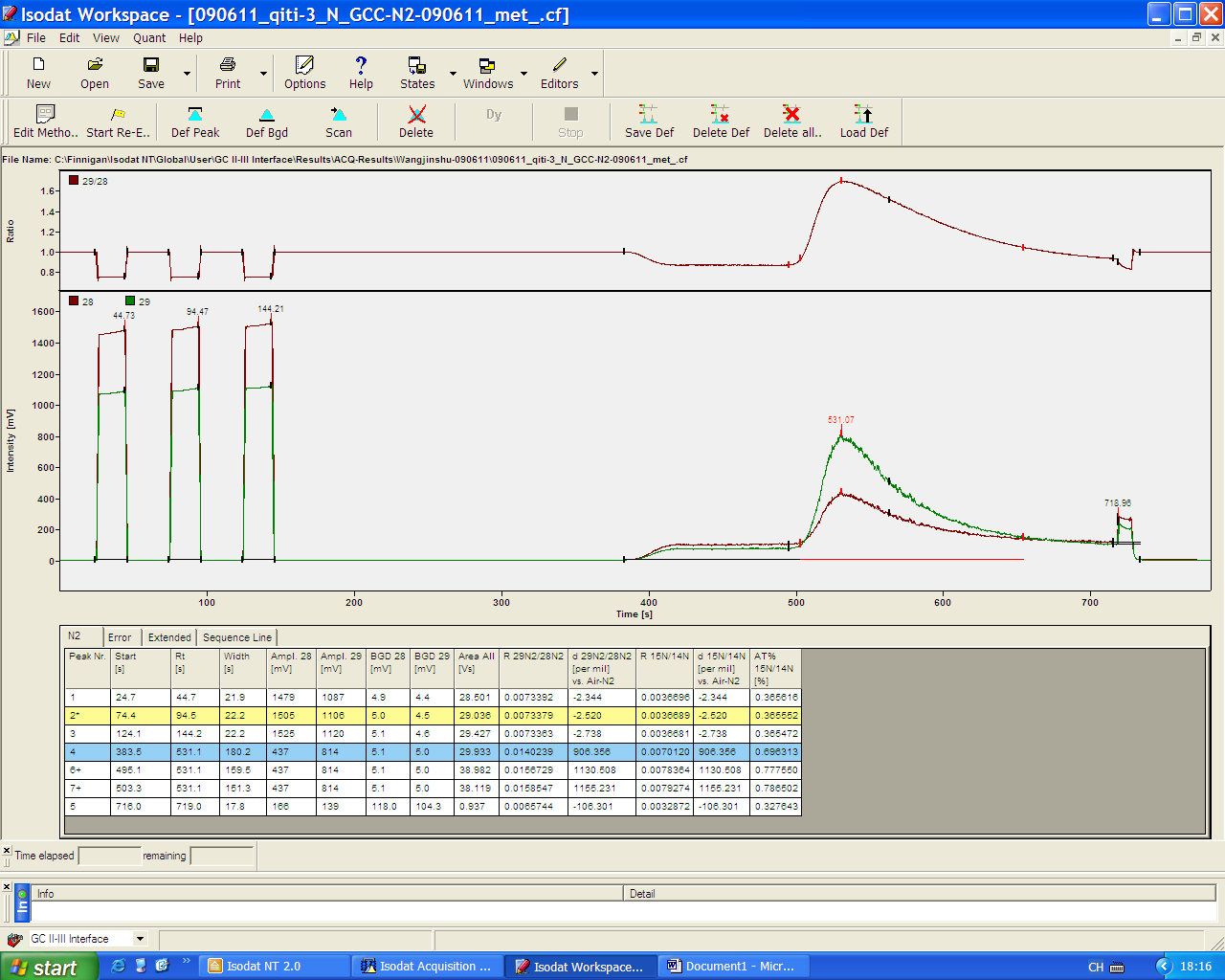
 **(a)**


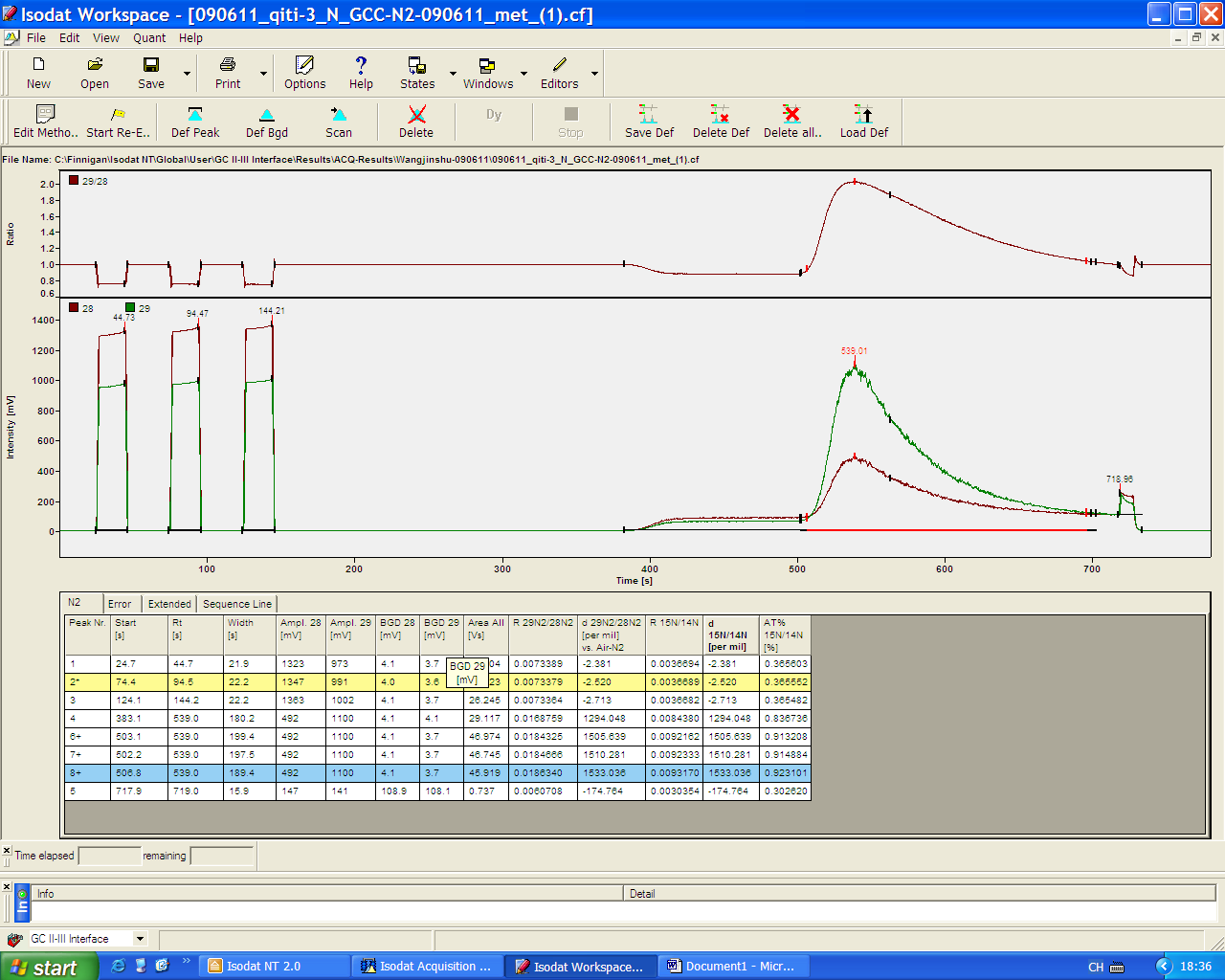
 **(b)**

Figure S5︱Mass spectroscopy detection result of denitrifying gaseous product (a) with ^15^NO_3_**^—^** addition and (b) with ^15^NO_2_**^—^** addition.

1. * Corresponding authors. Tel.: +86 22 84655498; Fax: +86 22 23328809.

   E-mail address: jingfengwang@hotmail.com [↑](#footnote-ref-2)
